# A Systematic Review and Meta-Analysis of Proteomics Literature on the Response of Human Skeletal Muscle to Obesity/Type 2 Diabetes Mellitus (T2DM) Versus Exercise Training

**DOI:** 10.3390/proteomes5040030

**Published:** 2017-11-11

**Authors:** Kanchana Srisawat, Sam O. Shepherd, Paulo J. Lisboa, Jatin G. Burniston

**Affiliations:** 1 Research Institute for Sport and Exercise Sciences, Liverpool John Moores University, Liverpool L3 3AF, UK; k.srisawat@2015.ljmu.ac.uk (K.S.); S.Shepherd@ljmu.ac.uk (S.O.S.); 2 Bureau of Non-Communicable Diseases, Department of Diseases Control, Ministry of Public Health, Nonthaburi 11000, Thailand; 3 Department of Applied Mathematics, Liverpool John Moores University, Liverpool L3 3AF, UK; P.J.Lisboa@ljmu.ac.uk

**Keywords:** type 2 diabetes, obese, insulin resistance, high intensity exercise, protein abundance, mass spectrometry, systematic review (SR), meta-analysis (MA), Preferred Reporting items for Systematic Review and Meta-analyses (PRISMA)

## Abstract

We performed a systematic review and meta-analysis of proteomics literature that reports human skeletal muscle responses in the context of either pathological decline associated with obesity/T2DM and physiological adaptations to exercise training. Literature was collected from PubMed and DOAJ databases following PRISMA guidelines using the search terms ‘proteom*’, and ‘skeletal muscle’ combined with either ‘obesity, insulin resistance, diabetes, impaired glucose tolerance’ or ‘exercise, training’. Eleven studies were included in the systematic review, and meta-analysis was performed on a sub-set (four studies) of the reviewed literature that reported the necessary primary data. The majority of proteins (*n* = 73) more abundant in the muscle of obese/T2DM individuals were unique to this group and not reported to be responsive to exercise training. The main response of skeletal muscle to exercise training was a greater abundance of proteins of the mitochondrial electron transport chain, tricarboxylic acid cycle and mitochondrial respiratory chain complex I assembly. In total, five proteins were less abundant in muscle of obese/T2DM individuals and were also reported to be more abundant in the muscle of endurance-trained individuals, suggesting one of the major mechanisms of exercise-induced protection against the deleterious effects of obesity/T2DM occurs at complex I of the electron transport chain.

## 1. Introduction

The incidence of obesity and type 2 diabetes (T2DM) has reached epidemic proportions and is a major burden on both individual patients and healthcare providers. Non-communicable diseases including metabolic syndrome and T2DM may be largely preventable through correct selection of lifestyle choices, and arguably the most important component of a healthy lifestyle is physical activity. For example, there is irrefutable evidence that low levels of physical activity or low cardiorespiratory fitness increase the risk of T2DM and Cardiovascular disease (CVD) [1]. In contrast, regular exercise confers a lower risk of mortality even in individuals that smoke or display other established CVD risk factors such as hypertension or dyslipidaemia [2].

Despite the robust link between exercise capacity and all-cause mortality the mechanisms responsible for the benefits of exercise are little understood. Nevertheless changes that occur in skeletal muscle are thought to be particularly important because this tissue represents approximately 40% of body mass and is responsible for both physical work and the majority (~80%) of insulin-stimulated glucose uptake [3]. Moreover, skeletal muscle exhibits profound plasticity in response to changes in environment including physical activity/inactivity and diet. Muscle is also easily accessible in humans, and there is currently a wealth of literature from hypothesis-led reductionist experiments reporting human muscle adaptation to exercise or diseases such as T2DM. Without disregarding the importance of these works, we have favoured the use of post-genomic hypothesis generating techniques, in particular proteomics. Our chosen approach is based on the premise that the proteome is a highly relevant level of study because it is the end product of genetic, epigenetic, and post-transcriptional processes, and the function of a cell/tissue is determined by its protein complement. Moreover, we regard the proteome as being the interface between the genome and the environment and is therefore ideally located for the study of complex polygenic processes that are responsive to environment factors. Last but not least, proteomic studies use comprehensive non-targeted analysis to generate data that can be interrogated by unsupervised statistical techniques and therefore have the potential to discover entirely new information.

We and others [4,5] have previously conducted narrative reviews of muscle proteomics literature from human studies investigating the benefits of exercise or the patho-biochemical changes that occur in obese individuals or T2DM patients. However, a major shortcoming of the previous reviews (including our own) is that the selection of articles by the reviewing authors could have introduced bias. This is not a trivial matter because it is entirely at odds with the unbiased hypothesis generating nature of proteomics and other -omic sciences. Moreover, because we do not fully understand the mechanisms responsible for the metabolic dysfunction of muscle or the preventive effects of regular exercise, we are not in a position to predict which proteins or processes are of greatest importance and therefore should be elevated above others when selecting future targets for hypothesis-led research.

In an attempt to address this issue, we have performed a systematic review of proteomics literature, thereby matching the non-targeted data collection of proteomics with the systematic/non-biased selection of research literature. Initially, our aim was to systematically review proteomic data relating to the effects of exercise in the muscle of humans that are obese, glucose intolerant or have T2DM. The subsequent literature search revealed that to date just one article [6] reported such data, whereas the majority of publications focused on either the pathophysiological adaptation associated with metabolic disorders/disease or the physiological adaptation to exercise training. Based on the rationale that some exercise-induced adaptations may directly oppose patho-biochemical changes associated with obesity and impaired glucose tolerance [7], we chose to systematically review each sub-area independently in an attempt to find contrasts that may indicate proteins common to each scenario. Therefore, the aim of this study was to review proteomic studies that have investigated human skeletal muscle in the context of either (i) pathological decline associated with obesity, insulin resistance, or T2DM; and (ii) physiological adaptations in response to exercise training. Specifically, we were driven by the questions, “What changes occur in the skeletal muscle proteome of humans that are obese, exhibit signs of the metabolic syndrome or diagnosed with T2DM?” and “What changes occur in the skeletal muscle proteome of humans that partake in regular exercise?”

## 2. Materials and Methods

### 2.1. Design and Delimitations of the Review

A systematic review was conducted according to the Preferred Reporting Items for Systematic Reviews and Meta-Analyses (PRISMA) guidelines [8]. Our overarching inclusion criteria were English full-text peer-reviewed articles that used proteomics methodologies to study protein abundances in human skeletal muscle. For the purposes of this review we define proteomics as non-targeted investigations at the protein level that use comprehensive/high-throughput analysis techniques to generate new information and hypotheses. Two sub-categories of literature were then investigated: research question 1 (RQ1) considered patho-physiological adaptations associated with obesity, impaired glucose tolerance (IGT), or T2DM; research question 2 (RQ2) considered physiological adaptations to exercise training. Literature reporting the effects of exercise was delimited to voluntary exercise including endurance training that primarily consists of a metabolic load/stimulus, or resistance exercise training that primarily relies on mechanical overload as the exercise stimulus. Body mass index (BMI) was used to stratify participants as normal weight (NW; BMI 18–24.9 kg/m^2^), overweight (OW; BMI 25–29.99 kg/m^2^), obese (OB; BMI 30–39.99 kg/m^2^) or morbidly obese (MOB; BMI > 40 kg/m^2^). Intermediate hyperglycaemia or IGT was defined as a fasting plasma glucose concentration of 6.1–6.9 mmol·L^−1^, whereas T2DM was defined as a fasting plasma glucose concentration of ≥7.0 mmol·L^−1^ or a 2 h glucose tolerance test plasma glucose concentration of ≥11.1 mmol·L^−1^ [9].

The methodological quality of the systematic review was assessed using checklists refined from the AMSTAR tool [10,11] and Delphi list [12].

### 2.2. Article Selection

Four stages of literature appraisal including identification, screening, eligibility, and inclusion were used. PubMed and the Directory of Open Access Journals (DOAJ) databases were searched using the following terms for RQ1: proteom* AND skeletal muscle AND obesity OR insulin resistance OR impaired glucose tolerance OR diabetes (RQ1); and for RQ2: proteom* AND skeletal muscle AND exercise. Searches were limited to the period between 1996 and 2016, and the last literature search was performed on 14 November 2016.

### 2.3. Data Extraction

The guidelines of the National Institute for Health and Care Excellence (NICE, UK) were used to structure our systematic review, and data was extracted from articles according to the PICO (Participant, Indicator, Comparator, Outcome) model. This method for developing and scoping systematic review questions was developed in accordance with the Cochrane collaboration systematic review database. In the current review, “Participants” were sub-set according to the definitions of RQ1 and RQ2, and “Indicators” such as BMI, glucose tolerance, and training status were extracted where relevant. The “Comparator” was health status (RQ1) or training status (RQ2), and the “Outcome” data collected were the identity and relative abundance of muscle proteins.

### 2.4. Meta-Analysis

Meta-analyses were conducted to assess the separate effects of either obesity/T2DM (RQ1) or exercise training (RQ2) on the abundance of muscle proteins. Descriptive and statistical data were extracted from each report and analysed according to the respective experiment design and research question. Effect size (ES) was calculated from mean ± SE data using Cohen’s difference score (*d*) [13] or *p*-value data retrieved from either independent [14] or dependent tests [15,16,17] was converted to Pearson’s product moment correlation (*r*). Note that all proteins were initially analysed separately, and then the combined ES for proteins reported across multiple experiments was calculated as the sum of z*_r_* divided by the square root of the number of studies (n), as described in Howitt and Cramer [18]. Both random and fixed effect models were applied to compute the summary effects following the method described by Rothstein et al. [19] and were analysed using Comprehensive Meta-analysis software version 2.0 (Biostat Inc., Englewood, NJ, USA). The effect of either disease or exercise intervention on individual protein abundance change were categorised into four groups; trivial (*r* < 0.10), small (0.30 ≥ *r* ≥ 0.10), medium (0.30 < *r* ≤ 0.5), and large (*r* > 0.5). All groups were categorised according to Cohen’s table of effect size magnitudes [20].

## 3. Results

### 3.1. RQ1 Article Selection

**Figure 1:**
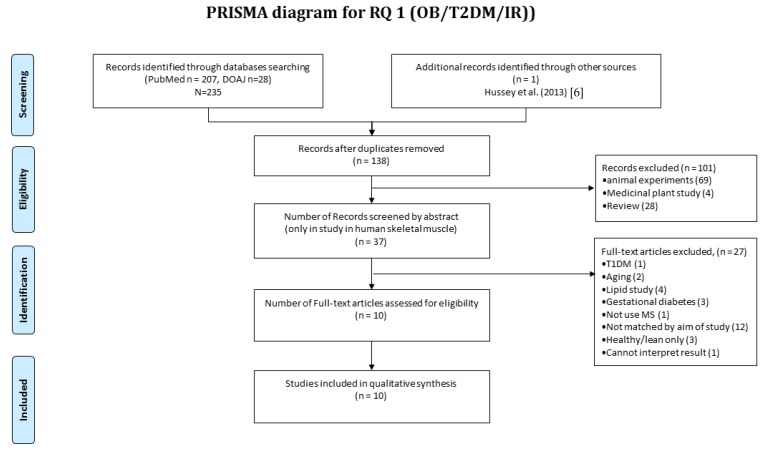
Systematic review of muscle proteomics data relating to human obesity and T2DM. A systematic review was conducted according to the PRISMA method. In total, 235 publications were collected in the initial searches and 138 were included after elimination of duplicates. Each abstract was screened against the eligibility criteria and a further 101 publications were excluded. The majority (*n* = 69) of the excluded publications reported data from animal models, while *n* = 28 were review articles and *n* = 4 reported the medicinal effects of plant extracts. Therefore, 37 full-text articles were assessed in detail and in all 10 publications were retained for outcome quality assessment using The Delphi List.

In total, 235 publications were collected during the initial searches and 138 were included after elimination of duplicates. Each abstract was screened against the eligibility criteria and a further 101 publications were excluded. The majority (*n* = 69) of the excluded publications reported data from animal models, whilst *n* = 28 were review articles, and *n* = 4 reported the medicinal effects of plant extracts. Therefore, 37 full-text articles were assessed in detail, and 10 publications were retained for further assessment (Figure 1). The earliest reported study meeting the eligibility criteria was Højlund et al. [21] published in 2003, and therefore the current review considers literature over a 13 year period from 2003 to 2016.

### 3.2. RQ1 Data Extraction

Table S1 summarises the content of articles relevant to RQ1 using the PICO system, which includes Population, Indicator, Comparison, and Outcome measures (Table S1). The population of participants in the literature reviewed was entirely of white Caucasian ethnicity. The number of participants ranged from 8 [22] to 10 [21] per study, and the total number of participants included in the current systematic review was *n* = 94 control and *n* = 182 case. Two publications reported co-morbidities such as pre-hypertension in obese participants [22] or hypertension amongst participants with T2DM [23]. BMI was the primary indicator used to define obesity and on average the BMI of participants in the case groups ranged from 30.2 to 57.3 kg·m^−2^ [14]. In all studies, age was matched between case and control groups. The age of control participants ranged from 33 [22] to 51 years [24,25], while obese participants were between 34 [22] and 50 years [24,25], and T2DM patients were between 43 [21] and 58 years [26]. Sex-specific differences have not been investigated in the proteomics literature to date. Hittel et al. [14] used females only and analysed samples of rectus abdominus, which is unique to this study. The majority of studies [21,22,24,26] recruited roughly equal numbers of males and females but in 2 two studies [23,25], the sex of the participants was not reported. The majority of studies [6,22,23,25,26,27] investigated biopsy samples obtained from the vastus lateralis. Højlund et al. [21] and Hussey et al. [6] report direct analysis of biopsy material and regenerated human myocytes, whereas three studies [23,25,27] were conducted entirely in human myotube cell cultures.

All outcome data were semi-quantitative, i.e., relative protein abundance. In total, the abundance of 156 proteins was reported to be statistically different between case (obese/T2DM) and control muscle (Table S2). There were 89 proteins that were more abundant in muscle of obese/T2DM patients and 3 (myosin heavy chain 2 (YH2), glutamine tRNA ligase (SYQ) and glycogen phosphorylase, (PYGM)) of these were repeated in more than one study. The most common biological processes amongst these proteins is the tricarboxylic acid (TCA) cycle, gluconeogenesis and muscle contraction. A total of 61 non-redundant proteins were less abundant in obese/T2DM patients, and only one protein (myosin regulatory light chain 2 ventricular cardiac muscle isoform, MLRV_HUMAN) was repeated in more than one study. Gene ontological analysis found biological process enriched in this group included muscle filament proteins, mitochondrial electron transport chain—NADH to ubiquinone, and mitochondrial ATP synthesis coupled proton transport.

### 3.3. RQ1 Meta-Analysis

**Figure 2:**
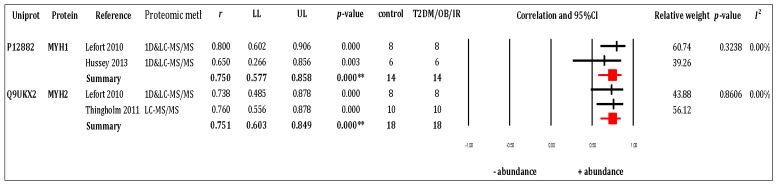
Forest plots of meta-analysis outcomes of muscle proteomics data relating to human obesity and T2DM. Two out of 13 proteins were significantly different (MYH1 and MYH2). Effect size (ES) of each protein was combined by two studies (*k* = 2) and was presented based on weighted Pearson product moment correlation (*r*) with 95% Confidence interval (95% CI). Both proteins were illustrated large effect size and were analysed by fixed-model ($r_{\mathrm{MYH}1}$ = 0.750 (0.577, 0.858), $p_{\mathrm{MYH}1}$ = 0.000, $I_{\mathrm{MYH}1}^{2}$ = 0.00%, *p* = 0.324; and $r_{\mathrm{MYH}2}$ = 0.751(0.603, 0.849), $p_{\mathrm{MYH}2}$ = 0.000, $I_{\mathrm{MYH}2}^{2}$ = 0.00%, *p* = 0.861).

Six [6,14,21,22,24,25] out of 11 studies included in the systematic review reported data in a format amenable to meta-analysis. The other five studies were excluded from the meta-analysis because they reported relative differences in protein abundance between groups but did not provide data regarding group averages or the distribution (i.e., SD or SEM or *p*-value) of values within each group. Moreover, numerous proteins were found significantly different in one study only and were therefore not applicable to meta-analysis. Thirteen proteins (ECH1, HBA, MLRS, MLRV, MYH1, MYH2, PYGM, G3P, NDUS2, ATP5F1, ATPB, COX2, and APOA1) were reported in more than one study. Two proteins (MYH1 and MYH2, which mainly function in muscle) exhibited statistical difference that revealed a large effect of disease on protein abundance. Forest plots of these proteins are presented in Figure 2.

### 3.4. RQ2 Article Selection

**Figure 3:**
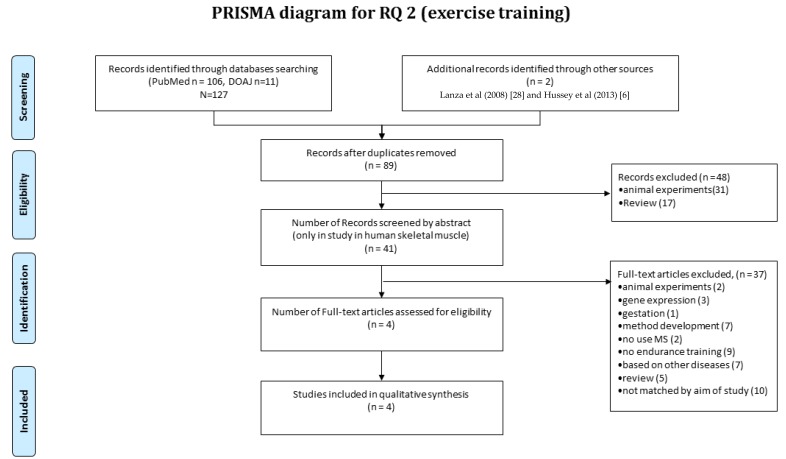
Systematic review of muscle proteomics data relating to human muscle responses to exercise. A systematic review was conducted according to the PRISMA method. In total, 127 publications were collected in the initial searches and one was manually integrated for screening. Then, 89 were included after elimination of duplicates. Each abstract was screened against the eligibility criteria, and a further 48 publications were excluded. The majority (*n* = 31) of the excluded publications conducted work in animal or were review articles (*n* = 17). Therefore, four full-text articles underwent quality assessment, and in all, four publications were retained for outcome quality assessment using the Delphi List.

The systematic review of muscle proteomics data relating to human muscle responses to exercise (i.e., RQ2) was separate to the review of literature relevant to RQ1. In total, 127 publications were collected in the initial searches, and two [6,28] were manually integrated for screening. Forty-one papers were included after elimination of duplicates, and when each abstract was screened against the eligibility criteria, a further 48 publications were excluded. The majority (*n* = 31) of the excluded studies were conducted without identifying the protein response to exercise or were performed in non-human animal models, and 17 were review articles (Figure 3). Therefore, four full-text articles passed the quality threshold and were retained for further assessment. The earliest studies meeting these criteria were Lanza et al. [28] reporting a cross-sectional comparison of habitually trained versus untrained individuals, and Holloway et al. [16] reporting longitudinal analysis in response to a six-week regimen of high-intensity interval training.

### 3.5. RQ2 Data Extraction

Table S2 summarises the content of articles relevant to RQ2 using the PICO system (Table S2). The number of participants in uncontrolled longitudinal trials [16,17] ranged between 5 [16] and 8 [17], whereas randomised controlled studies [6,15,28] used between 5 and 10 in each group. The total number of participants included in the current systematic review was 77 (*n* = 32 control, *n* = 32 case and *n* = 13 in pre- vs post-exercise interventions). These studies included both males and females [6,28], males only [16,17], or did not report the sex of their participants [15]. Three publications [6,16,17] report the effects of an endurance exercise intervention, whereas two [15,28] reported cross-sectional analysis of trained versus untrained populations. The endurance exercise interventions comprised of training programmes that used cycling (80%VO_2max_ without session duration reported, 7 day/week for 14 days) [17], or a four-week intervention involving a combination of continuous moderate-intensity cycling (55% VO_2max_, 60 min, 3 day/week) and high intensity intermittent cycling (70%VO_2max_, 6 × 5 min, 2 day/week) [6], or treadmill running for six weeks (6 × 1 min intervals at 90–100% VO_2max_, 3 day/week) [16].

Comparisons were made between trained and untrained individuals using either a cross-sectional design [15,28] or within-subject longitudinal designs that were either uncontrolled [16,17,29] or randomised controlled trials [6]. Consistent with RQ1, all outcome data were semi-quantitative, i.e., relative protein abundance. In total, the abundance of 75 proteins was reported to be statistically different between exercise trained and untrained muscle (Table S2).

Sixty-eight non-redundant proteins were more abundant in muscle of exercise-trained individuals, and two proteins (ATP synthase subunits alpha and beta, ATPA and ATPB) were repeated in more than one study. The most common biological processes included the tricarboxylic acid cycle, mitochondrial electron transport chain—NADH to ubiquinone, mitochondrial respiratory chain complex I assembly, gluconeogenesis. Seven proteins were less abundant in exercise-trained muscle, and one protein (alpha B crystallin, CYRAB) was reported by more than pne publication.

### 3.6. RQ2 Meta-Analysis

**Figure 4:**
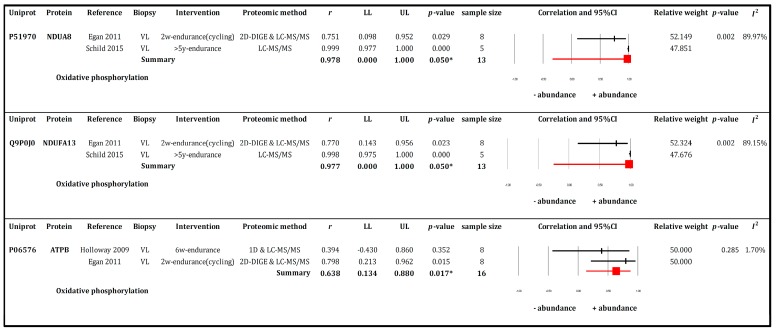
Forest plots of meta-analysis outcomes of muscle proteomics data relating to human muscle responses to endurance exercise. The summary effect of 3 individual proteins and abundance change were analysed by Mixed-model method and depicted in pink rectangular box. All proteins function mainly in the oxidative phosphorylation pathway. The effect size (ES) of each protein was combined for two studies (*k* = 2) (Holloway 2009, Egan 2011, or Schild 2015) and was presented based on weighted Pearson product moment correlation (*r*) with 95% Confidence interval (95% CI). All proteins large effect size and presented from trivial to very large range when refer to population (95% CI) i.e., $r_{\mathrm{NDUA}8}$ = 0.978 (0.000, 1.000), $p_{\mathrm{NDUA}8}$ = 0.050, $I_{\mathrm{NDUA}8}^{2}$ = 89.97%, $r_{\mathrm{NDUA}13}$ = 0.977 (0.000, 1.000), $p_{\mathrm{NDUA}13}$ = 0.050, $I_{\mathrm{NDUA}13}^{2}$ = 89.15%, and $r_{\mathrm{ATPB}}$ = 0.638(0.134, 0.880), $p_{\mathrm{ATPB}}$ = 0.017, $I_{\mathrm{ATPB}}^{2}$ = 1.70%.

Meta-analysis was conducted on data collected from longitudinal studies [15,16,17] that investigated the effects of exercise training. Meta-analysis was not performed on data from cross-sectional studies because Lanza et al. [28] did not report group averages or the distribution (i.e., SD or SEM) of values within each group, which left only Schild et al. [15]. Five proteins (CRYAB, ATPA, NDUA8, NDUFA13, and ATPB) were reported in more than one study. Among these, three proteins were found to be statistically different and are presented in Figure 4.

### 3.7. Internal Quality Assessment

Publications were scored using the criteria of the Delphi list [12]. The quality of evidence presented in the 11 articles collected under RQ1 was ranked as medium to high (62.5–75.0%). Evidence collected under RQ2 was sub-divided in to cross-sectional (case versus control) or longitudinal (within subject) designs. The longitudinal studies were ranked medium high, whereas the case-control studies were ranked high.

Using the AMSTAR evaluation method [10,11], the quality of our current systematic review was ranked ‘high’ (81.2%). The score for our current work was less than 100% because it was not possible to conduct meta-analysis on data from all studies and, secondly, because we did not set specific criteria for handling grey literature.

## 4. Discussion

We have systematically reviewed proteomics literature on human muscle in the context of either (i) the pathophysiological decline associated with obesity, insulin resistance or type 2 diabetes mellitus or (ii) physiological adaptations in response to exercise training. Our systematic review and meta-analyses highlight a shift toward a fast-twitch myofibre profile and a decrease in the abundance of electron transport chain components, particularly NADH-ubiquinone subunits of complex I, in the muscle of obese and T2DM individuals. In contrast, the main response of the muscle proteome to exercise training was a greater abundance of proteins of the mitochondrial electron transport chain, tricarboxylic acid cycle and mitochondrial respiratory chain complex I assembly. Although comparatively few proteins were shared across the two different systematic review questions, RQ1 and RQ2, our systematic review is consistent with the paradigm that some exercise-induced adaptations may directly oppose patho-biochemical changes associated with obesity/impaired glucose tolerance/T2DM.

**Figure 5:**
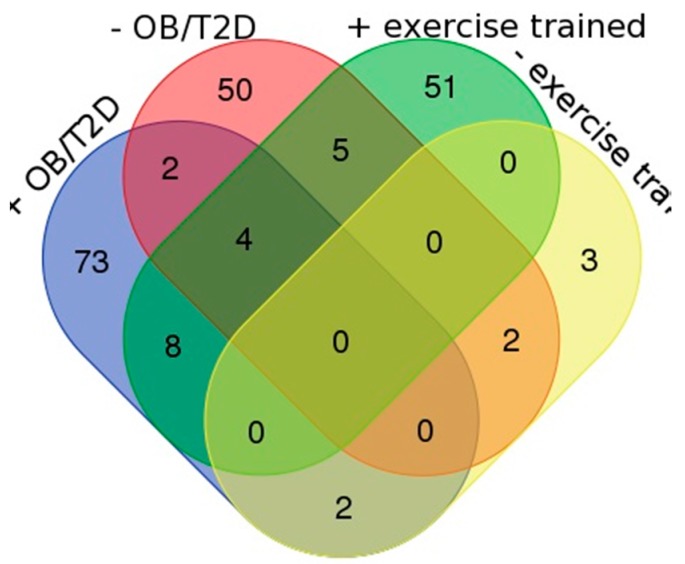
Venn diagram of non-redundant proteins extracted by systematic review of literature relating to RQ1 and RQ2. Proteins reported under RQ1 to be greater in abundance in the muscle of obese/T2DM muscle (+OB/TSDM, *n* = 73) or less abundant in the muscle of obese/T2DM muscle (−OB/T2DM, *n* = 50) are overlaid with proteins reported under RQ2 that were either more abundant in exercise-trained muscle (+EXERC, *n* = 51) or less abundant in exercise trained muscle (−EXERC, *n =* 3).

Figure 5 presents a Venn diagram showing the number of significantly different proteins that were reported within each research question. The greatest number (*n* = 73) of proteins was reported to be specifically more abundant in the muscle of obese/T2DM patients (Figure 5 and Table S1). Gene ontology analysis discovered significant (Fisher’s with BH corrected *p* < 0.05) enrichment of the biological process phrases “muscle filament sliding” (MYH2, 3, 8 MYL1, and TNNT3) and “protein stabilisation” (CDC37, CCt4, CCT8, GAPDH, HSP90AB1, and PIK3R1) amongst this group. In addition, there was significant enrichment of the KEGG pathways “biosynthesis of amino acids” and “PI3K-Akt signalling” that are conspicuous features of the wider literature relating to the patho-biochemical adaptation of muscle in T2DM patients. Individual proteins within the highlighted PI3K-Akt signalling pathway, such as tenascin-C, may constitute interesting targets for follow-up studies.

Tenascin C is a glycoprotein of the extracellular matrix that is associated with the molecular signature of tissue injury [30]. Typically, the expression of tenascin-C is low or undetectable in healthy tissues but transiently increases in response to injury, including damaging eccentric exercise of skeletal muscle [31]. Tenascin-C was initially identified as a toll-like receptor 4 (TLR4) agonist that mediates the sustained local inflammatory response associated with arthritic joint disease [32]. Subsequently tenascin-C has also been implicated in the chronic low-grade inflammation associated with obesity. Catalán et al. [33] reports tenascin-C gene expression is significantly elevated in visceral adipose tissue (but not subcutaneous adipose tissue) of obese humans, and elevated levels of tenascin-C expression also occur in the adipose tissue of animal models of either genetic or diet-induced obesity. Similarly, increases in hepatic tenascin-C expression occur in obese individuals with non-alcoholic fatty liver disease (NAFLD) [34], and in each of these scenarios tenascin-C interacts with TLR4 to augment local tissue inflammation. It should be noted that our review found that tenascin C was upregulated in cultured myotubes and not ‘adult’ skeletal muscle, and therefore future studies should investigate tenascin C in skeletal muscle directly. This is important because chronic inflammation in obese individuals is associated with muscle insulin resistance, and it could be hypothesised that the tenascin-C/TLR4 signalling axis is also involved in this response.

We compared the outcomes of each review question by investigating proteins that are common or distinct between the case and control groups of RQ1 and RQ2 (Figure 5). In exercise-trained individuals, 51 proteins were specifically more abundant and biological process phrases enriched in this group included mitochondrial electron transport, NADH to ubiquinone, tricarboxylic acid cycle, and mitochondrial respiratory chain complex I assembly. This profile shared close similarity to the biological process phases that were enriched amongst the 50 proteins that were less abundant in muscle of obese/T2DM individuals, which included metabolic proteins and specifically enzymes of the electron transport chain. However, when compared at the level of individual proteins just five proteins were shared between these groups. Proteins that were less abundant in obese/T2DM muscle but more abundant in exercised muscle include: NADH ubiquinone oxidoreductase subunits A8 (NDUA8), B8 (NDUB8) and S2 (NDUS2); glutamic-oxaloacetic transaminase 2 (AATM) and ATP synthase beta (ATPB). Therefore, a difference in the abundance of subunits of complex I (NADH ubiquinone oxidoreductase) is one of the primary hypotheses generated from our systematic review of proteomics literature. In contrast, Fast-twitch myofibrillar proteins, MLRS, and SERCA1 were more abundant in muscle of obese individuals and less abundant in exercise trained muscle.

Currently, Hussey et al. [6] is the only proteomic work to directly investigate exercise responses in T2DM muscle and reports a dominant increase in the abundance of enzymes of the malate-aspartate shuttle which serves to transport NADH from the cytosol to within the mitochondria. This adaptation is a long established effect of endurance exercise in the muscle of healthy human participants [35]. However, Hussey et al. [6] did not detect any change in the abundance of complex I subunits in T2DM patients. The question arises whether this is because the ability to change the abundance of complex I components is lost/diminished in obesity/T2DM, or if the study was not capable of detecting this effect. Hussey et al. [6] reports GeLC-MS/MS analysis, which achieves a good depth of proteome coverage, but differential analysis of protein abundances was performed by spectral counting, which can suffer from missing data that makes comparative analyses difficult. In addition, the exercise intervention was relatively short (a four-week duration), and whilst there was a trend for improved glucose tolerance, no statistically significant improvement in fasting glucose or insulin values, OGTT, or Matsuda index was reported [6]. Therefore, targeted analysis is required in the future to resolve whether defects in the abundance and assembly of complex I subunits are a key contributing factor to the patho-biochemical adaptation of muscle in T2DM patients.

Within the systematic reviews, eight proteins were reported to be more abundant in both obese/T2DM and exercise-trained muscle. These proteins included 2 enzymes of the TCA cycle (citrate synthase, CISY and acontiase, ACON) and enzymes involved in glycolysis (phosphoglycerate mutase 2, PGAM2), fatty acid oxidation (acyl-CoA dehydrogenase, very long chain, ACADVL), and the electron transport chain (succinate dehydrogenase complex flavoprotein subunit A, SDHA) in addition to Tu translation elongation factor, mitochondrial, EFTU and methylcrotonoyl-CoA carboxylase 2, MCCB, which contributes to the catabolism of branched chain amino acids leucine and valine.

We also performed a meta-analysis to increase the precision of the results obtained from the systematic review. Meta-analysis of data related to RQ1 (“pathological decline associated with obesity, insulin resistance or type 2 diabetes mellitus”) revealed a significant increase in the abundance of fast-twitch myosin heavy chain isoforms (MYH1 and MYH2) in obesity and T2DM. Three myosin isoforms are typically expressed in human muscle: slow-twitch type I (MYH7) and fast-twitch isoforms IIa (MYH2) and IIx (MYH1). The proportion of each isoform differs as a consequence of genetic background and habitual activity level and might typically be expected to be 50–60% type I, 20–30% IIa and 10–20% IIx [36]. A greater abundance of MYH1 and MYH2 is consistent with the higher proportion of fast twitch fibres and the lower oxidative capacity of skeletal muscle often observed in obese and T2DM individuals [6,37].

In contrast, meta-analysis of data collected under RQ2 (“physiological adaptations in response to exercise training”) identified just three proteins (NDUA8, NDUFA13, and ATPB) that were significantly more abundant after endurance exercise training. ATPB is a subunit of complex V and NDUA8 and NDUFA13 are responsible for subunit I of the mitochondrial respiratory chain. The reported upregulation of these OXPHOS proteins in response to endurance-type exercise training signifies an improved capacity for oxidative ATP production.

We have conducted systematic reviews and meta-analyses to consolidate knowledge from the existing proteomics literature relating to the effects of obesity and type 2 diabetes or exercise training on human skeletal muscle. Because our work used hypothesis generating proteomics data our results highlight questions for future research rather than conclusive outcomes at the population level. We have been able to select targets and generate hypotheses from more robust data than is available from each of the individual studies conducted so far. Nonetheless, our work was necessarily limited by the small number and breadth of the current proteomics literature, and in particular few papers reported data amenable to meta-analysis. In addition, the rapid pace of technical development in the field of proteomics over the past decade has resulted in relatively poor equivalency of data across the current body of literature. For example, contrary to our hypothesis, we do not report widespread and diametrically opposite responses between the muscle of obese and type 2 diabetics compared exercise-trained individuals. This could be an indication that the underlying mechanisms are so complex that changes in the proteins in response to training are different to the proteins that change in response to disease yet lead to diametric yet opposing whole-body responses (e.g., increase or decrease in insulin sensitivity), or it could be an artefact caused by the wide variety of proteomics techniques that have been applied in this area. In particular, it can be confusing to compare 2D gel data that resolves proteins to their constituent species or proteoforms with LC-MS/MS profiling data that reports the overall abundance of all species of each protein.

Until recently, differential analysis of muscle using LC-MS was challenging and in our previous narrative review [38] we advocated the continued use of 2D gel-based proteomics because this suffers less from issues caused by missing data that occurs in LC-MS/MS work flows, including differential labelling and spectra counting techniques. More recently we [39] reported label-free LC-MS profiling of skeletal muscle using data-independent acquisition and Progenesis Quantitative Informatics for Proteomics, which enabled high-throughput and comprehensive analysis of the skeletal muscle proteome. In the future, more wide spread application of robust platforms such as this will generate more parallel data with greater equivalency of proteome coverage across studies from different laboratories. In addition to this technological advance, our systematic review also highlights a number of areas for improvement in experiment design. In the proteomics literature to date, few studies report the medical history or full medical characteristics of the participants. For example, the majority of literature did not include information regarding hypertension or other cardiovascular risk factors such as chronic kidney disease that are common co-morbidities in obese/type 2 diabetic patients. The literature reviewed herein generally used equal numbers of male and female participants but as yet sexual dimorphism in the muscle response to obesity or exercise training has not been specifically investigated. In addition, ethnic backgrounds other than white Caucasian need to be investigated, especially in light of epidemiological evidence that Asian and Polynesian populations amongst others are at a greater risk of T2DM than white Caucasians [40].

## 5. Conclusions

In conclusion, skeletal muscle of obese and T2DM individuals is marked by a shift toward a fast-twitch myofibre profile and a decrease in the abundance of glycolytic enzymes and electron transport chain components, particularly NADH-ubiquinone subunits of complex I. A handful of proteins associated with fatty acid or amino acid metabolism and the tricarboxylic acid cycle were more abundant in both obese/T2DM patients and exercise-trained individuals, which may be consistent with the paradigm known as the athlete’s paradox. The majority of proteins (*n* = 73) that are significantly more abundant in the muscle of obese/T2DM individuals were unique to this group and were not reported to be responsive to exercise training. Functional annotation of these proteins found enrichment of the biological process “protein stabilisation” and the PI3K-Akt pathway. Amongst the proteins listed in the PI3K-Akt pathway was tenascin C, which may represent an important target for more focused follow-up studies in muscle. The main response of skeletal muscle to exercise training was a greater abundance of proteins of the mitochondrial electron transport chain, tricarboxylic acid cycle and mitochondrial respiratory chain complex I assembly. In total, three proteins were less abundant in muscle of obese/T2DM individuals and were also reported to be more abundant in the muscle of endurance-trained individuals suggesting one of the major mechanisms of exercise-induced protection against the deleterious effects of obesity/glucose intolerance and T2DM occurs at complex I of the electron transport chain.

## Supplementary Materials

The following are available online www.mdpi.com/2227-7382/5/4/30/s1. Table S1: Summarises the main content (RQ1) of each article according to the PICO principle. Table S2: Summarises the main content (RQ2) of each article according to the PICO principle. Table S3: List of proteins in accordance with Venn diagram (Figure 5).
