# Supplementary material for: A Systematic Review and Meta-Analysis of Proteomics Literature on the Response of Human Skeletal Muscle to Obesity/Type 2 Diabetes Mellitus (T2DM) Versus Exercise Training
Source: Proteomes. 2017 Nov 11;5(4):30. doi: 10.3390/proteomes5040030 (PMC5748565; doi:10.3390/proteomes5040030)
Supplement: Supplementary file 1 [file proteomes-05-00030-s001.zip › supplementary table 1 for proteomes.pdf]

Supplementary Table 1 summarises the main content (RQ1) of each article according to the PICO principle, which includes Population, Indicator, Comparison and Outcome measures. The earliest reported study meeting the eligability criteria was HØjlund et al. published in 2003 (Hojlund et al. 2003), therefore the current review considers literature over a 12 year study period from 2003 to 2016.
Note-OB is obesity, MOB is Morbidly obese, T2DM is type 2 diabetes mellitus, HT is hypertension, OWHLT is overweight with healthy, LEHLT is lean with healthy, LEOW is lean with slightly overweight, NGT is non-glucose tolerance, OCs is Obese-control
***presented significant difference at p-value <0.05 or under**

Reference	Participants (M/F)	Study groups			Outcome measures		
		Case	Control	Method in proteomics	Uniprot accession number		Protein name (gene name)*
HØjlund et al.	N=15 (8/7)	T2DM	OWHLT	2D-DIGE	Up-regulation (6)		
2003	Vastus lateralis (biopsy & cell)	n = 9 (5/4)	n = 6 (3/3)	pH 4-7	P08238	Heat shock protein HSP 90-beta	HSP90AB1 (HSP90AB1 HSP90B, HSPC2, HSPCB)
		age 45 ± 2 y	age 46 ± 2 y		P11021	78 kDa glucose-regulated protein	GRP78 (HSPA5 GRP78)
		BMI 33.3± 1.9 kg.m^{-2}	BMI 25.7 ± 1.2 kg.m^{-2}	MALDI-TOF (P-32)	P12109	Collagen alpha-1(VI) chain	COL6A1 (COL6A1)
					P12277	Creatine kinase B-type	KCRB (CKB CKBB)
					P36871	Phosphoglucomutase-1	PGM1 (PGM1)
					Q96A32	Myosin regulatory light chain 2, skeletal muscle isoform	MLRS (MYLPF)
					Down-regulation (2)		
					P06576	ATP synthase subunit beta, mitochondrial	ATPB (ATP5B)
					P10916	Myosin regulatory light chain 2, ventricular/cardiac muscle isoform	MLRV
Hittel et al.	N=18 (0/18)	OW/OB	LEHLT	2D-DIGE			
2005		n = 6 (0/6)	n = 6	pH 3-10 11 cm	Up-regulation (3)		
	Rectus abdominus	age 44 ± 3 y	age 45 ± 3 y				
		BMI 30.2 ± 0.81 kg.m^{-2}	BMI 23.8 ± 0.58 kg.m^{-2}	MALDI-TOF			
					P00568	Adenylate kinase isoenzyme 1	KAD1 (AK1)
		MOB			P04075	Fructose-bisphosphate aldolase A	ALDOA (ALDOA ALDA)
		n = 6 (0/6)			P04406	Glyceraldehyde-3-phosphate dehydrogenase	G3P (GAPDH GAPD, CDABP0047, OK/SW-cl.12)
		age 38 ± 3 y			Down-regulation (0)		
		BMI 53.8 ± 3.5 kg.m^{-2}					
Lefort et al.	N = 34 (18/16)	OB (with pre-HT/pre-DM)	LEOW (with pre-HT)	1D-SDS-PAGE (12%)	Up-regulation (10)		
2010		n = 14 (7/7)	n = 20 (11/9)	HPLC-MS/MS	A6NNS2	Dehydrogenase/reductase SDR family member 7C	DRS7C (DHRS7C SDR32C2)
	Vastus lateralis	age 38 ± 4 y	age 38 ± 3 y		O14983	Sarcoplasmic/endoplasmic reticulum calcium ATPase 1	AT2A1 (SERCA1)
		BMI 36 ± 1 kg.m^{-2}	BMI 25 ± 1 kg.m^{-2}		P02647	Apolipoprotein A-I	APOA1 (APOA1)
					P11217	Glycogen phosphorylase, muscle form	PYGM (PYGM)
					P12882	Myosin-1	MYH1 (MYH1)
					P27105	Erythrocyte band 7 integral membrane protein	STOM (STOM)
					P32119	Peroxiredoxin-2	PRDX2 (Prdx2 Tdpx1, Tpx)
					P54289	Voltage-dependent calcium channel subunit alpha-2/delta-1	CA2D1 (CACNA2D1 CACNL2A, CCHL2A, MHS3)
					Q9UHG3	Prenylcysteine oxidase 1	PCYOX (PCYOX1 KIAA0908, PCL1, UNQ597/PRO1183)
					Q9UKX2	Myosin-2	MYH2 (MYH2 MYHSA2)
					Down-regulation (11)		
					E9PQ53	NADH dehydrogenase [ubiquinone] 1 subunit C2, isoform 2	NDUCR (NDUFC2-KCTD14)
					O14958	Calsequestrin-2	CASQ2 (CASQ2)
					O75306	NADH dehydrogenase [ubiquinone] iron-sulfur protein 2, mitochondrial	NDUS2 (NDUFS2)
					O95169	NADH dehydrogenase [ubiquinone] 1 beta subcomplex subunit 8, mitochondrial	NDUB8 (NDUFB8)
					P05166	Propionyl-CoA carboxylase beta chain, mitochondrial	PCCB (PCCB)
					P10606	Cytochrome c oxidase subunit 5B, mitochondrial	COX5B (COX5B)
					P51970	NADH dehydrogenase [ubiquinone] 1 alpha subcomplex subunit 8	NDUA8 (NDUFA8)
					Q02252	Methylmalonate-semialdehyde dehydrogenase [acylating], mitochondrial	MMSA (ALDH6A1 MMSDH)
					Q6ZMU5	Tripartite motif-containing protein 72	TRI72 (TRIM72 MG53)
					Q86Y39	NADH dehydrogenase [ubiquinone] 1 alpha subcomplex subunit 11	NDUAB (NDUFA11)
					Q92523	Carnitine O-palmitoyltransferase 1, muscle isoform	CPT1B
Hwang et al.	N = 24 (12/12)	OB (with pre-DM)	LEHLT	1D-SDS-PAGE	Up-regulation (9)		
2010		n = 8 (4/4)	n= 8 (4/4)	(4-20%)	O94760	N(G),N(G)-dimethylarginine dimethylaminohydrolase 1	DDAH1 (DDAH1 DDAH)
	Vastus lateralis	age 44 ± 3 y	age 37 ± 4 y	HPLC-MS/MS	P30101	Protein disulfide-isomerase A3	PDIA3 (PDIA3 ERP57, ERP60, GRP58)
		BMI 31.6 ± 0.8 kg.m^{-2}	BMI 24.3 ± 0.8 kg.m^{-2}		P47897	Glutamine--tRNA ligase	SYQ (QARS)
					P49720	Proteasome subunit beta type-3	PSB3 (PSMB3)

		T2DM			P50990	T-complex protein 1 subunit theta	TCPQ (CCT8 C21orf112, CCTQ, KIAA0002)
		n= 8 (4/4)			P50991	T-complex protein 1 subunit delta	TCPD (CCT4 CCTD, SRB)
		age 48 ± 3 y			Q16543	Hsp90 co-chaperone Cdc37	CDC37 (CDC37 CDC37A)
		BMI 29.0 ± 1.1 $kg.m^{-2}$			Q93034	Cullin-5	CUL5 (CUL5 VACM1)
					Q9Y2K3	Myosin-15	MYH15 (MYH15 KIAA1000)
					Down-regulation (5)		
						Cytochrome c oxidase subunit 6C (Cytochrome c oxidase polypeptide VIc)	COX6C (COX6C)
					P14927	Cytochrome b-c1 complex subunit 7	QCR7 (UQCRB UQBP)
					P35609	Alpha-actinin-2	ACTN2 (ACTN2)
					Q9NP98	Myozenin-1	MYOZ1
					Q9NX63	MICOS complex subunit MIC19	MIC19 (CHCHD3 MIC19, MINOS3)
Thingholm et al	N=30	OB	LEHLT		Up-regulation (6)		
2011		n =10	n=10		P05413	Fatty acid-binding protein, heart	FABPH (FABP3 FABP11, MDGI)
	Vastus lateralis (myotube cell)	age 49 ± 1 y	age 51 ± 1 y		P11055	Myosin-3	MYH3 (MYH3)
		BMI 33.7 ± 1.4 $kg.m^{-2}$	BMI 24.2 ± 0.5 $kg.m^{-2}$		P13535	Myosin-8	MYH8 (MYH8)
					P24821	Tenascin	TENA (TNC)
		OBT2DM			P60842	Eukaryotic initiation factor 4A-I	IF4A1 (EIF4A1)
		n=10			Q9UKX2	Myosin-2	MYH2 (MYH2 MYHSA2)
		age 50 ± 1 y		nLC-MS/MS (iTRAQ)			
		BMI 33.5 ± 1.1 $kg.m^{-2}$			Down-regulation (6)		
					O14558	Heat shock protein beta-6	HSPB6 (HSPB6)
					P00325	Alcohol dehydrogenase 1B	ADHB1 (ADH1B ADH2)
					P00813	Adenosine deaminase	ADA (ADA ADA1)
					P02647	Apolipoprotein A-I	APOA1 (APOA1)
					P40121	Macrophage-capping protein	CAPG (CAPG AFCP, MCP)
					P69905	Hemoglobin subunit alpha	HBA (HBA1 HBA2)
Giebelstein et al	N=31 (17/14)	OB (pre-DM)	LEHLT	2D-DIGE	Up-regulation (20)		
2012		n=11 (6/5)	n=10 (5/5)	LC-MS/MS (CyDye)	O75323	Protein NipSnap homolog 2	NIPS2 (GBAS NIPSNAP2)
	Vastus lateralis	age 49 ± 1 y	age 51 ± 1 y		P02679	Fibrinogen gamma chain	FIBG (FGG PRO2061)
		BMI 33.7 ± 1.4 $kg.m^{-2}$	BMI 24.2 ± 0.5 $kg.m^{-2}$		P04406	Glyceraldehyde-3-phosphate dehydrogenase	G3P (GAPDH GAPD, CDABP0047, OK/SW-cl.12)
					P05976	Myosin light chain 1/3, skeletal muscle isoform	MYL1
		OBT2DM			P06732	Creatine kinase M-type	KCRM (KCRM)
		n=10 (6/4)			P11217	Glycogen phosphorylase, muscle form	PYGM (PYGM)
		age 50 ± 1 y			P13929	Beta-enolase	ENOB (ENO3)
		BMI 33.5 ± 1.1 $kg.m^{-2}$			P14618	Pyruvate kinase PKM	KYPM (PKM OIP3, PK2, PK3, PKM2)
					P15259	Phosphoglycerate mutase 2	PGAM2 (PGAM2 PGAMM)
					P40925	Malate dehydrogenase, cytoplasmic	MDHC (MDH1, MDHA)
					P45378	Troponin T, fast skeletal muscle	TNNT3 (TNNT3)
					P54652	Heat shock-related 70 kDa protein 2	HSP72 (HSPA2)
					P55084	Trifunctional enzyme subunit beta, mitochondrial	ECHB (HADHB MSTP029)
					P63267	Actin, gamma-enteric smooth muscle	ACTH (ACTG2 ACTA3, ACTL3, ACTSG)
					P69905	Hemoglobin subunit alpha	HBA (HBA1 HBA2)
					Q13011	Delta(3,5)-Delta(2,4)-dienoyl-CoA isomerase, mitochondrial	ECH1
					Q14469	Transcription factor HES-1	HES1 (HES1 BHLHB39, HL, HRY)
					Q96A32	Myosin regulatory light chain 2, skeletal muscle isoform	MLRS (MYLPF)
					Q9H7C9	Mth938 domain-containing protein	AAMDC (AAMDC C11orf67, PTD015)
					Q9Y281	Cofilin-2	COF2 (CFL2)
					Down-regulation (6)		
					P08590	Myosin light chain 3	MYL3 (MYL3)
					P10916	Myosin regulatory light chain 2, ventricular/cardiac muscle isoform	MLRV
					P13805	Troponin T, slow skeletal muscle	TNNT1 (TNNT1 TNT)
					P17661	Desmin	DESM (DES)
					P40123	Adenylyl cyclase-associated protein 2	CAP2 (CAP2)
					P62736	Actin, aortic smooth muscle	ACTA (ACTA2 ACTSA, ACTVS, GIG46)

Al-Khalili et al 2014a	N=20 (na/na) Vastus lateralis (cell line)	T2DM n=10	NGT n=10	2D-DIGE pH 3-11 24cm LC-MS/MS (CyDye)	Up-regulation (8)		
					P04792	Heat shock protein beta-1	HSPB1 (HSPB1 HSP27, HSP28)
					P25786	Proteasome subunit alpha type-1	PSA1 (PSMA1 HC2, NU, PROS30, PSC2)
					P25789	Proteasome subunit alpha type-4	PSA4 (PSMA4 HC9, PSC9)
					P49721	Proteasome subunit beta type-2	PSB2 (PSMB2)
					P60900	Proteasome subunit alpha type-6	PSA6 (PSMA6)
					P62195	26S protease regulatory subunit 8	PRS8 (PSMC5 SUG1)
					Q9BTZ2	Dehydrogenase/reductase SDR family member 4	DHRS4 (DHRS4 SDR25C2, UNQ851/PRO1800)
					Q9UBQ0	Vacuolar protein sorting-associated protein 29	VPS29 (VPS29 DC15, DC7, MDS007)
					Down-regulation (3)		
					O75083	WD repeat-containing protein 1	WDR1 (WDR1)
					P14866	Heterogeneous nuclear ribonucleoprotein L	HNRPL (HNRNPL HNRPL, P/OKcl.14)
					Q13838	Spliceosome RNA helicase DDX39B	DX39B (DDX39B BAT1, UAP56)
Al-Khalili et al 2014b	N=20 (20/0) Vastus lateralis (cell line)	T2DM (10/0)	NGT (10/0)	2D-DIGE pH 3-11 24cm LC-MS/MS (CyDye)	Up-regulation (29)		
					O75390	Citrate synthase, mitochondrial	CISY (CS)
					O75534	Cold shock domain-containing protein E1	CSDE1 (CSDE1 D1S155E, KIAA0885, NRU, UNR)
					O96008	Mitochondrial import receptor subunit TOM40 homolog	TOM40 (TOMM40 C19orf1, PEREC1, TOM40)
					P13010	X-ray repair cross-complementing protein 5	XRCC5 (XRCC5 G22P2)
					P14061	Estradiol 17-beta-dehydrogenase 1	DHB1 (HSD17B1 E17KSR, EDH17B1, EDH17B2, EDHB17, SDR28C1)
					P14866	Heterogeneous nuclear ribonucleoprotein L	HNRPL (HNRNPL HNRPL, P/OKcl.14)
					P23786	Carnitine O-palmitoyltransferase 2, mitochondrial	CPT2 (CPT2 CPT1)
					P25705	ATP synthase subunit alpha, mitochondrial	ATPA (ATP5A1 ATP5A, ATP5AL2, ATPM)
					P30044	Peroxiredoxin-5, mitochondrial	PRDX5 (PRDX5 ACR1, SBBI10)
					P31040	Succinate dehydrogenase [ubiquinone] flavoprotein subunit, mitochondrial	SDHA
					P34897	Serine hydroxymethyltransferase, mitochondrial	GLYM (SHMT2)
					P38117	Electron transfer flavoprotein subunit beta	ETFB (ETFB FP585)
					P40926	Malate dehydrogenase, mitochondrial	MDHM (MDH2)
					P42765	3-ketoacyl-CoA thiolase, mitochondrial	THIM (ACAA2)
					P47897	Glutamine--tRNA ligase	SYQ (QARS)
					P49411	Elongation factor Tu, mitochondria	EFTU (TUFM)
					P49748	Very long-chain specific acyl-CoA dehydrogenase, mitochondrial	ACADV (ACADVL)
					P50213	Isocitrate dehydrogenase [NAD] subunit alpha, mitochondrial	IDH3A (IDH3A)
					P83111	Serine beta-lactamase-like protein LACTB, mitochondrial	LACTB (LACTB MRPL56, UNQ843/PRO1781)
					Q02809	Procollagen-lysine,2-oxoglutarate 5-dioxygenase 1	PLOD1 (PLOD1 LLH, PLOD)
					Q12906	Interleukin enhancer-binding factor 3	ILF3 (ILF3 DRBF, MPHOSPH4, NF90)
					Q12931	Heat shock protein 75 kDa, mitochondrial	TRAP1 (TRAP1 HSP75)
					Q13263	Transcription intermediary factor 1-beta	TIF1B (TRIM28 KAP1, RNF96, TIF1B)
					Q14191	Werner syndrome ATP-dependent helicase	WRN (WRN RECQ3, RECQL2)
					Q92499	ATP-dependent RNA helicase DDX1	DDX1 (DDX1)
					Q92945	Far upstream element-binding protein 2	FUBP2 (KHSRP FUBP2)
					Q99798	Aconitate hydratase, mitochondrial	ACON (ACO2)
					Q9HCC0	Methylcrotonoyl-CoA carboxylase beta chain, mitochondrial	MCCB (MCCC2)
					Q9NSD9	Phenylalanine--tRNA ligase beta subunit	SYFB (FARSB FARSLB, FRSB, HSPC173)
					Down-regulation (18)		
					P04792	Heat shock protein beta-1	HSPB1 (HSPB1 HSP27, HSP28)
					P06733	Alpha-enolase	ENOA (ENO1 ENO1L1, MBPB1, MPB1)
					P07900	Heat shock protein HSP 90-alpha	HSP90A (HSP90AA1 HSP90A, HSPC1, HSPCA)
					P09211	Glutathione S-transferase P	GSTP1 (GSTP1 FAEES3, GST3)
					P13797	Plastin-3	PLST (PLS3)
					P16152	Carbonyl reductase [NADPH] 1	CBR1 (CBR1)
					P28161	Glutathione S-transferase Mu 2	GSTM2 (GSTM2 GST4)
					P30084	Enoyl-CoA hydratase, mitochondrial	ECHM (ECHS1)
					P30711	Glutathione S-transferase theta-1	GSTT1 (GSTT1)

					P35080	Profilin-2	PROF2 (PFN2)
					P60981	Destrin	DEST (DSTN ACTDP, DSN)
					P61086	Ubiquitin-conjugating enzyme E2 K	UBE2K (UBE2K HIP2, LIG)
					P62937	Peptidyl-prolyl cis-trans isomerase A	PPIA (PPIA CYPA)
					P82279	Protein crumbs homolog 1	CRUM1 (CRB1)
					Q16555	Dihydropyrimidinase-related protein 2	DPYL2 (DPYSL2 CRMP2, ULIP2)
					Q99497	Protein deglycase DJ-1	PARK7
					Q9H299	SH3 domain-binding glutamic acid-rich-like protein 3	SH3L3 (SH3BGRL3 P1725)
					Q9Y617	Phosphoserine aminotransferase	SERC (PSAT1 PSA)
Caruso et al	N=22 (14/8)	OCs (pre-DM)	LCs	1D-SDS-PAGE	Up-regulation (9)		
2014		n=7 (5/2)	n=8 (5/3)	HPLC-MS/MS	O14732	Inositol monophosphatase 2	IMPA2 (IMPA2 IMP.18P)
	Vastus lateralis biopsy	age 44 ± 4 y	age 46 ± 4 y		P19474	E3 ubiquitin-protein ligase TRIM21	RO52 (TRIM21 RNF81, RO52, SSA1)
		BMI 32.5 ± 1.4 kg.m^{-2}	BMI 23.2 ± 0.5 kg.m^{-2}		P21980	Protein-glutamine gamma-glutamyltransferase 2	TGM2 (TGM2)
					P27986	Phosphatidylinositol 3-kinase regulatory subunit alpha	P85A (PIK3R1 GRB1)
		T2DM			P30153	Serine/threonine-protein phosphatase 2A 65 kDa regulatory subunit A alpha isoform	2AAA (PPP2R1A)
		n=7 (4/3)			P62753	40S ribosomal protein S6	RS6 (RPS6 OK/SW-cl.2)
		age 53 ± 4 y			P62993	Growth factor receptor-bound protein 2	GRB2 (GRB2 ASH)
		BMI 32.8 ± 1.5 kg.m^{-2}			P67775	Serine/threonine-protein phosphatase 2A catalytic subunit alpha isoform	PP2AA (PPP2CA/B)
					Q13061	Triadin	TRDN (TRDN)
					Down-regulation (4)		
					O75083	WD repeat-containing protein 1	WDR1 (WDR1)
					P05091	Aldehyde dehydrogenase, mitochondrial	ALDH2 (ALDH2 ALDM)
					P33176	Kinesin-1 heavy chain	KINH (KIF5B KNS, KNS1)
					Q92901	60S ribosomal protein L3-like	RL3L (RPL3L)
Hussey	N = 12 (8/4) at rest	T2DM	Control	1D-SDS-PAGE (4-20%)	Up-regulation (2)		
2013		n = 6 (4/2)	n = 6 (4/2)		P02765	Alpha-2-HS-glycoprotein	FETUA (AHSG FETUA, PRO2743)
	Vastus lateralis	age 54 ± 4 y	age 48 ± 2 y	HPLC-MS/MS	P02787	Serotransferrin	TF (TRFE)
		BMI 29 ± 2 kg.m^{-2}	BMI 28 ± 3		Down-regulation (13)		
			kg.m^{-2}		P00403	Cytochrome c oxidase subunit 2	COX2 (MT-CO2 COII, COXII, MTCO2)
					P00505	Aspartate aminotransferase, mitochondrial	AATM (GOT2)
					P12882	Myosin-1	MYH1 (MYH1)
					P15121	Aldose reductase	ALDR (AKR1B1 ALDR1)
					P17174	Aspartate aminotransferase, cytoplasmic	AATC (GOT1)
					P24539	ATP synthase F(0) complex subunit B1, mitochondrial	ATP5F1 (ATP5F1)
					P25705	ATP synthase subunit alpha, mitochondrial	ATPA (ATP5A1 ATP5A, ATP5AL2, ATPM)
					P40926	Malate dehydrogenase, mitochondrial	MDHM (MDH2)
					Q13011	Delta(3,5)-Delta(2,4)-dienoyl-CoA isomerase, mitochondrial	ECH1
					Q7Z4W1	L-xylulose reductase	DCXR (DCXR SDR20C1)
					Q8N335	Glycerol-3-phosphate dehydrogenase 1-like protein	GPD1L (GPD1L KIAA0089)
					Q99623	Prohibitin-2	PHB2 (PHB2 BAP, REA)
					Q9UDW1	Cytochrome b-c1 complex subunit 9	UCR9 (UQCR10 UCRC, HSPC119)
