# Supplementary material for: A Systematic Review and Meta-Analysis of Proteomics Literature on the Response of Human Skeletal Muscle to Obesity/Type 2 Diabetes Mellitus (T2DM) Versus Exercise Training
Source: Proteomes. 2017 Nov 11;5(4):30. doi: 10.3390/proteomes5040030 (PMC5748565; doi:10.3390/proteomes5040030)
Supplement: Supplementary file 1 [file proteomes-05-00030-s001.zip › supplementary table 2 for proteomes.pdf]

Supplementary Table 2 summarises the main content (RQ2) of each article according to the PICO principle, which includes Population, Intervention, Comparison and Outcome measures. The earliest reported study meeting the eligability criteria was done by Holloway et al (2009), therefore the current review considers literature over a 7 year study period from 2009 to 2015.
Note-OB is obesity, MOB is Morbidly obese, T2DM is type 2 diabetes mellitus, HT is hypertension, OWHLT is overweight with healthy, LEHLT is lean with healthy, LEOW is lean with slightly overweight, NGT is non-glucose tolerance, OCs is Obese-control
***presented significant difference at p-value <0.05 or under**

		Study groups		Intervention					Outcome measure		
Reference	Participants (M/F)	Case	Control	Modulity	Intensity	Volume (min/session)	Frequency (times/week)	Duration (week)		Protein description	
**Holloway 2009**	N = 5 (5/0)	One group		Endurance	6x1 min	30	3	6	Up-regulation (4)		
		Pre-post		(motorised threadmill)	90-100% V_{O2max}				P25705	ATP synthase subunit alpha, mitochondrial	ATPA (ATP5A1 ATP5A, ATP5AL2, ATPM)
	age 21 ± 2 y								P06576	ATP synthase subunit beta, mitochondrial	ATPB (ATP5B)
					4 min				P31040	Succinate dehydrogenase [ubiquinone] flavoprotein subunit, mitochondrial	SDHA
	Vastus lateralis				50% V_{O2max}				P40939	Trifunctional enzyme subunit alpha, mitochondrial	ECHA (HADHA HADH)
	(biopsy)										
	1D-SDS-PAGE										
	LC-MS/MS										
**Egan 2011**	N = 8 (8/0)	One group		Endurance training	80% V_{O2max}	60	everyday	2	Up-regulation (21)		
	Age 23 ± 2 y	Pre-post		(cycling)				(14 d)	P03915	NADH-ubiquinone oxidoreductase chain 5	NU5M (MT-ND5 MTND5, NADH5, ND5)
	BMI 23.6 ± 0.9 kg.m-2								P51970	NADH dehydrogenase [ubiquinone] 1 alpha subcomplex subunit 8	NDUA8 (NDUFA8)
									Q9P0J0	NADH dehydrogenase [ubiquinone] 1 alpha subcomplex subunit 13	NDUFA13 (NDUFA13 GRIM19, CDA016, CGI-39)
	Vastus lateralis								P25705	ATP synthase subunit alpha, mitochondrial	ATPA (ATP5A1 ATP5A, ATP5AL2, ATPM)
	(biopsy)								P00505	Aspartate aminotransferase, mitochondrial	AATM (GOT2)
	2DIGE								P02144	Myoglobin	MYG (MB)
	LC-MS/MS								P04075	Fructose-bisphosphate aldolase A	ALDOA (ALDOA ALDA)
									P04179	Superoxide dismutase [Mn], mitochondrial	SODM (SOD2)
									P06576	ATP synthase subunit beta, mitochondrial	ATPB (ATP5B)
									P07954	Fumarate hydratase, mitochondrial	FUMH (FH)
									P09622	Dihydrolipoyl dehydrogenase, mitochondrial	DLDH (DLD GCSL, LAD, PHE3)
									P15259	Phosphoglycerate mutase 2	PGAM2 (PGAM2 PGAMM)
									P17540	Creatine kinase S-type, mitochondrial	KCRS (CKMT2)
									P29590	Protein PML (Promyelocytic leukemia protein) (RING finger protein 71) (Tripartite motif-containing protein 19)	PML (PML MYL, PP8675, RNF71, TRIM19)
									P30086	Phosphatidylethanolamine-binding protein 1	PEBP1 (PEBP1 PBP, PEBP)
									P36957	Dihydrolipoyllysine-residue succinyltransferase component of 2-oxoglutarate dehydrogenase complex, mitochondrial	ODO2 (DLST)
									P40926	Malate dehydrogenase, mitochondrial	MDHM (MDH2)
									P49411	Elongation factor Tu, mitochondria	EFTU (TUFM)
									P60174	Triosephosphate isomerase	TPIS (TPI1, TPI)
									P61604	10 kDa heat shock protein, mitochondrial	CH10 (HSPE1)
									Q9UIJ7	GTP:AMP phosphotransferase AK3, mitochondrial	KAD3 (AK3)
									Down-regulation (5)		
									P20674	Cytochrome c oxidase subunit 5A, mitochondrial	COX5A (COX5A)
									P02511	Alpha-crystallin B chain	CRYAB (CRYAB CRYA2, HSPB5)
									O14983	Sarcoplasmic/endoplasmic reticulum calcium ATPase 1	AT2A1 (SERCA1)
									Q96A32	Myosin regulatory light chain 2, skeletal muscle isoform	MLRS (MYLPF)
									P11762	Galectin-1	LEG1 (LGALS1)
**Husssey 2013**	N = 12 (8/4)	One group		Endurance	60 min	60	3 d/wk	4	Up-regulation (3)		
		Pre-post			55% V_{O2max}				P02765	AHSG cDNA FLJ55606, higly similar to Alpha-2-HS-glycoprotein	FETUA (AHSG, FETUA, PRO2743)
	T2DM								O95169	NADH dehydrogenase [ubiquinone] 1 beta subcomplex subunit 8, mitochondrial	NDUB8 (NDUFB8)
	(OW/OB)				6x5 min	30	2 d/wk		P12882	Myosin-1	MYH1 (MYH1)
	n = 6 (4/2)				70% V_{O2max}						
									Down-regulation (3)		
	age 54 ± 4 y								P02511	Alpha-crystallin B chain	CRYAB (CRYAB)
									P15121	Aldose reductase	ALDR (AKR1B1 ALDR1)
	BMI 29 ± 2								Q8N335	Glycerol-3-phosphate dehydrogenase 1-like protein	GPD1L (GPD1L KIAA0089)
	$kg.m^{-2}$										
	Vastus lateralis										
	(biopsy)										
	1D-SDS-PAGE										
	LC-MS/MS										
**Schild 2015**	N = 10	ET & LE	UT & LE/OW/OB	Endurance exercice	na	5h per week		>5 y	Up-regulation (43)		
									O00330	Pyruvate dehydrogenase protein X component, mitochondrial	ODPX (PDHX PDX1)
	Vastus leteralis		n = 5						O43181	NADH dehydrogenase [ubiquinone] iron-sulfur protein 4, mitochondrial	NDUS4 (NDUFS4)
	(biopsy)	n = 5	Age 24 ± 5 y						O75306	NADH dehydrogenase [ubiquinone] iron-sulfur protein 2, mitochondrial	NDUS2 (NDUFS2)
		Age 27 ± 2 y							O75489	NADH dehydrogenase [ubiquinone] iron-sulfur protein 3, mitochondrial	NDUS3 (NDUFS3)
			BMI 25.7 ± 5.0						O75746	Calcium-binding mitochondrial carrier protein Aralar1	CMC1 (SLC25A12 ARALAR1)
	LC-MS/MS	BMI 23.4 ± 1.6 $kg.m^{-2}$	$kg.m^{-2}$						O75947	ATP synthase subunit d, mitochondrial	ATP5H (ATP5H My032)
									P00403	Cytochrome c oxidase subunit 2	COX2 (MT-CO2 COII, COXII, MTCO2)
									P03915	NADH-ubiquinone oxidoreductase chain 5	NU5M (MT-ND5 MTND5, NADH5, ND5)
									P06576	ATP synthase subunit beta, mitochondrial	ATPB (ATP5B)
									P11177	Pyruvate dehydrogenase E1 component subunit beta, mitochondrial	ODPB (PDHB PHE1B)
									P12235	ADP/ATP translocase 1	ADT1 (SLC25A4 ANT1)
									P14854	Cytochrome c oxidase subunit 6B1	COX6B1 (COX6B1 COX6B)
									P17540	Creatine kinase S-type, mitochondrial	KCRS (CKMT2)
									P21912	Succinate dehydrogenase [ubiquinone] iron-sulfur subunit, mitochondrial	SDHB (SDHB SDH, SDH1)
									P24310	Cytochrome c oxidase subunit 7A1, mitochondrial	COX7A1 (COX7A1 COX7AH)
									P24539	ATP synthase F(0) complex subunit B1, mitochondrial	ATP5F1 (ATP5F1)
									P28331	NADH-ubiquinone oxidoreductase 75 kDa subunit, mitochondrial	NDUS1 (NDUFS1)
									P30038	Delta-1-pyrroline-5-carboxylate dehydrogenase, mitochondrial	AL4A1 (ALDH4A1 ALDH4, P5CDH)
									P31040	Succinate dehydrogenase [ubiquinone] flavoprotein subunit, mitochondrial	SDHA

P36542	ATP synthase subunit gamma, mitochondrial (F-ATPase gamma subunit)	ATPG (ATP5C1 ATP5C, ATP5CL1)
P48735	Isocitrate dehydrogenase [NADP], mitochondrial	IDHP (IDH2)
P49748	Very long-chain specific acyl-CoA dehydrogenase, mitochondrial	ACADV (ACADVL)
P49753	Acyl-coenzyme A thioesterase 2, mitochondrial	ACOT2
P54819	Adenylate kinase 2, mitochondrial	KAD2 (AK2)
Q00325	Phosphate carrier protein, mitochondrial	MPCP (SLC25A3 PHC, OK/SW-cl.48)
Q02218	2-oxoglutarate dehydrogenase, mitochondrial	ODO1 (OGDH)
Q02978	Mitochondrial 2-oxoglutarate/malate carrier protein	M2OM (SLC25A11 SLC20A4)
Q16795	NADH dehydrogenase [ubiquinone] 1 alpha subcomplex subunit 9, mitochondrial	NDUA9 (NDUFA9 NDUFS2L)
Q96IX5	Up-regulated during skeletal muscle growth protein 5	USMG5 (USMG5 DAPIT, HCVFTP2, PD04912)
Q99643	Succinate dehydrogenase cytochrome b560 subunit, mitochondrial	C560 (SDHC CYB560, SDH3)
Q9HCC0	Methylcrotonoyl-CoA carboxylase beta chain, mitochondrial	MCCB (MCCC2)
Q9P2R7	Succinyl-CoA ligase [ADP-forming] subunit beta, mitochondrial	SUCB1 (SUCLA2)
Q9Y2Z9	Ubiquinone biosynthesis monooxygenase COQ6, mitochondrial	COQ6 (COQ6 CGI-10)
Q9Y6C9	Mitochondrial carrier homolog 2	MTCH2 (MTCH2 MIMP, HSPC032)
Q9Y6M9	NADH dehydrogenase [ubiquinone] 1 beta subcomplex subunit 9	NDUB9 (NDUFB9 LYRM3, UQOR22)
P31930	Cytochrome b-c1 complex subunit 1, mitochondrial	QCR1 (UQCRC1)
O75964	ATP synthase subunit g, mitochondrial	ATP5L (ATP5L)
O75390	Citrate synthase, mitochondrial	CISY (CS)
Q99798	Aconitate hydratase, mitochondrial	ACON (ACO2)
P42126	Enoyl-CoA delta isomerase 1, mitochondrial	ECI1 (ECI1 DCI)
Q13011	Delta(3,5)-Delta(2,4)-dienoyl-CoA isomerase, mitochondrial	ECH1
Q53EW7	Branched-chain-amino-acid aminotransferase	Q53EW7 (BCAT2)
O75208	Ubiquinone biosynthesis protein COQ9, mitochondrial	COQ9 (COQ9)
