# Supplementary material for: A Systematic Review and Meta-Analysis of Proteomics Literature on the Response of Human Skeletal Muscle to Obesity/Type 2 Diabetes Mellitus (T2DM) Versus Exercise Training
Source: Proteomes. 2017 Nov 11;5(4):30. doi: 10.3390/proteomes5040030 (PMC5748565; doi:10.3390/proteomes5040030)
Supplement: Supplementary file 1 [file proteomes-05-00030-s001.zip › supplementary Venn (pooled).pdf]

+ in OB/T2DM	Function	Uniprot	Description	
	Adipogenesis	Q9H7C9	Mth938 domain-containing protein	AAMDC (AAMDC C11orf67, PTD015)
	Amino acid biosynthesis	P34897	Serine hydroxymethyltransferase, mitochondrial	GLYM (SHMT2)
	Antioxidant	P30044	Peroxiredoxin-5, mitochondrial	PRDX5 (PRDX5 ACR1, SBBI10)
	Antioxidant	P32119	Peroxiredoxin-2	PRDX2 (Prdx2 Tdpx1, Tpx)
	Binding protein (ATP, NT)	Q9NSD9	Phenylalanine--tRNA ligase beta subunit	SYFB (FARSB FARSLB, FRSB, HSPC173)
	Binding protein (ATP,DNA,NT,RNA)	Q92499	ATP-dependent RNA helicase DDX1	DDX1 (DDX1)
	Binding protein (Ca2+)	P21980	Protein-glutamine gamma-glutamyltransferase 2	TGM2 (TGM2)
	Binding protein (Ca2+)	P54289	Voltage-dependent calcium channel subunit alpha-2/delta-1	CA2D1 (CACNA2D1 CACNL2A, CCHL2A, MHS3)
	Binding protein (DNA,ATP,NT)	P13010	X-ray repair cross-complementing protein 5	XRCC5 (XRCC5 G22P2)
	Binding protein (DNA,RNA)	Q92945	Far upstream element-binding protein 2	FUBP2 (KHSRP FUBP2)
	Binding protein (Fe2+,Fe3+)	P02787	Serotransferrin	TF (TRFE)
	Binding protein (RNA)	O75534	Cold shock domain-containing protein E1	CSDE1 (CSDE1 D1S155E, KIAA0885, NRU, UNR)
	Binding protein (RNA)	P14866	Heterogeneous nuclear ribonucleoprotein L	HNRPL (HNRNPL HNRPL, P/OKcl.14)
	Binding protein (RNA)	P27105	Erythrocyte band 7 integral membrane protein	STOM (STOM)
	Binding protein (RNA)	P62753	40S ribosomal protein S6	RS6 (RPS6 OK/SW-cl.2)
	Chromosome rearrangement	P30153	Serine/threonine-protein phosphatase 2A 65 kDa regulatory subunit A alpha isoform	2AAA (PPP2R1A)
	Chromosome rearrangement	Q13263	Transcription intermediary factor 1-beta	TIF1B (TRIM28 KAP1, RNF96, TIF1B)
	DNA repair	Q14191	Werner syndrome ATP-dependent helicase	WRN (WRN RECQ3, RECQL2)
	ECM	P02679	Fibrinogen gamma chain	FIBG (FGG PRO2061)
	ECM	P12109	Collagen alpha-1(VI) chain	COL6A1 (COL6A1)
	ECM	P24821	Tenascin	TENA (TNC)
	ECM	Q02809	Procollagen-lysine,2-oxoglutarate 5-dioxygenase 1	PLOD1 (PLOD1 LLH, PLOD)
	ECM	P06732	Creatine kinase M-type	KCRM (KCRM)
	ECM	Q99623	Prohibitin-2	PHB2 (PHB2)
	Glucose metabolism	P04406	Glyceraldehyde-3-phosphate dehydrogenase	G3P (GAPDH GAPD, CDABP0047, OK/SW-cl.12)
	Glucose metabolism	P13929	Beta-enolase	ENOB (ENO3)
	Glucose metabolism	P14618	Pyruvate kinase PKM	KYPM (PKM OIP3, PK2, PK3, PKM2)
	Glucose metabolism	P36871	Phosphoglucomutase-1	PGM1 (PGM1)
	Immune response	P27986	Phosphatidylinositol 3-kinase regulatory subunit alpha	P85A (PIK3R1 GRB1)
	Immune response	P60842	Eukaryotic initiation factor 4A-I	IF4A1 (EIF4A1)
	Immune response	P62993	Growth factor receptor-bound protein 2	GRB2 (GRB2 ASH)
	Immune response	Q12906	Interleukin enhancer-binding factor 3	ILF3 (ILF3 DRBF, MPHOSPH4, NF90)
	Lipid metabolism	P05413	Fatty acid-binding protein, heart	FABPH (FABP3 FABP11, MDGI)
	Lipid metabolism	P14061	Estradiol 17-beta-dehydrogenase 1	DHB1 (HSD17B1 E17KSR, EDH17B1, EDH17B2, EDHB17, SDR28C1)
	Lipid metabolism	P23786	Carnitine O-palmitoyltransferase 2, mitochondrial	CPT2 (CPT2 CPT1)
	Lipid metabolism	P55084	Trifunctional enzyme subunit beta, mitochondrial	ECHB (HADHB MSTP029)
	Lysosome	Q9UHG3	Prenylcysteine oxidase 1	PCYOX (PCYOX1 KIAA0908, PCL1, UNQ597/PRO1183)
	Muscle	P05976	Myosin light chain 1/3, skeletal muscle isoform	MYL1
	Muscle	P11055	Myosin-3	MYH3 (MYH3)
	Muscle	P11217	Glycogen phosphorylase, muscle form	PYGM (PYGM)
	Muscle	P13535	Myosin-8	MYH8 (MYH8)
	Muscle	P45378	Troponin T, fast skeletal muscle	TNNT3 (TNNT3)
	Muscle	P63267	Actin, gamma-enteric smooth muscle	ACTH (ACTG2 ACTA3, ACTL3, ACTSG)
	Muscle	Q13061	Triadin	TRDN (TRDN)
	Muscle	Q9UKX2	Myosin-2	MYH2 (MYH2 MYHSA2)
	Muscle	Q9Y2K3	Myosin-15	MYH15 (MYH15 KIAA1000)
	Muscle	Q96A32	Myosin regulatory light chain 2, skeletal muscle isoform	MLRS (MYLPF)
	OXPHOS	O94760	N(G),N(G)-dimethylarginine dimethylaminohydrolase 1	DDAH1 (DDAH1 DDAH)
	OXPHOS	P38117	Electron transfer flavoprotein subunit beta	ETFB (ETFB FP585)
	OXPHOS	P06576	ATP synthase subunit beta, mitochondrial	ATPB (ATP5B)
	Porin	O96008	Mitochondrial import receptor subunit TOM40 homolog	TOM40 (TOMM40 C19orf1, PEREC1, TOM40)
	Protein biosynthesis	P47897	Glutamine--tRNA ligase	SYQ (QARS)
	Protein biosynthesis	P67775	Serine/threonine-protein phosphatase 2A catalytic subunit alpha isoform	PP2AA (PPP2CA/B)
	Protein rearrangement	P30101	Protein disulfide-isomerase A3	PDIA3 (PDIA3 ERP57, ERP60, GRP58)
	Repressor	Q14469	Transcription factor HES-1	HES1 (HES1 BHLHB39, HL, HRY)
	Skeletal muscle specific protein	P83111	Serine beta-lactamase-like protein LACTB, mitochondrial	LACTB (LACTB MRPL56, UNQ843/PRO1781)
	Stress reponse	O14558	Heat shock protein beta-6	HSPB6 (HSPB6)
	Stress reponse	P08238	Heat shock protein HSP 90-beta	HSP90AB1 (HSP90AB1 HSP90B, HSPC2, HSPCB)
	Stress reponse	P11021	78 kDa glucose-regulated protein	GRP78 (HSPA5 GRP78)
	Stress reponse	P19474	E3 ubiquitin-protein ligase TRIM21	RO52 (TRIM21 RNF81, RO52, SSA1)
	Stress reponse	P42765	3-ketoacyl-CoA thiolase, mitochondrial	THIM (ACAA2)
	Stress reponse	P49720	Proteasome subunit beta type-3	PSB3 (PSMB3)
	Stress reponse	P50990	T-complex protein 1 subunit theta	TCPQ (CCT8 C21orf112, CCTQ, KIAA0002)
	Stress reponse	P50991	T-complex protein 1 subunit delta	TCPD (CCT4 CCTD, SRB)
	Stress reponse	P54652	Heat shock-related 70 kDa protein 2	HSP72 (HSPA2)
	Stress reponse	Q12931	Heat shock protein 75 kDa, mitochondrial	TRAP1 (TRAP1 HSP75)
	Stress reponse	Q16543	Hsp90 co-chaperone Cdc37	CDC37 (CDC37 CDC37A)
	Structure	Q93034	Cullin-5	CUL5 (CUL5 VACM1)
	Structure	Q9Y281	Cofilin-2	COF2 (CFL2)
	TCA	O75323	Protein NipSnap homolog 2	NIPS2 (GBAS NIPSNAP2)
	TCA	P40925	Malate dehydrogenase, cytoplasmic	MDHC (MDH1, MDHA)
	TCA	P50213	Isocitrate dehydrogenase [NAD] subunit alpha, mitochondrial	IDH3A (IDH3A)
	Visual involvement	A6NNS2	Dehydrogenase/reductase SDR family member 7C	DRS7C (DHRS7C SDR32C2)

- in OB/T2DM	Function	Uniprot	Description	
	Structure	O75083	WD repeat-containing protein 1	WDR1 (WDR1)
	Structure	Q92901	60S ribosomal protein L3-like	RL3L (RPL3L)
	Structure	Q9H299	SH3 domain-binding glutamic acid-rich-like protein 3	SH3L3 (SH3BGRL3 P1725)
	Structure	Q9NP98	Myozenin-1	MYOZ1
	Structure	P40123	Adenylyl cyclase-associated protein 2	CAP2 (CAP2)
	Structure	P17661	Desmin	DESM (DES)
	Structure	P60981	Destrin	DEST (DSTN ACTDP, DSN)
	Structure	P35080	Profilin-2	PROF2 (PFN2)
	Stress reponse	O14558	Heat shock protein beta-6	HSPB6 (HSPB6)
	Stress reponse	Q99497	Protein deglycase DJ-1	PARK7
	Stress reponse	P07900	Heat shock protein HSP 90-alpha	HSP90A (HSP90AA1 HSP90A, HSPC1, HSPCA)
	Stress reponse	P61086	Ubiquitin-conjugating enzyme E2 K	UBE2K (UBE2K HIP2, LIG)
	Stress reponse	P04792	Heat shock protein beta-1	HSPB1 (HSPB1 HSP27, HSP28)
	Repressor	Q9NX63	MICOS complex subunit MIC19	MIC19 (CHCHD3 MIC19, MINOS3)
	Prevent neurodegeneration	P09211	Glutathione S-transferase P	GSTP1 (GSTP1 FAEES3, GST3)
	Prevent neurodegeneration	P28161	Glutathione S-transferase Mu 2	GSTM2 (GSTM2 GST4)
	Prevent neurodegeneration	P16152	Carbonyl reductase 1	
	Prevent neurodegeneration	P30711	Glutathione S-transferase theta-1	GSTT1 (GSTT1)
	OXPHOS	E9PQ53	NADH dehydrogenase [ubiquinone] 1 subunit C2, isoform 2	NDUCR (NDUFC2-KCTD14)
	OXPHOS	Q86Y39	NADH dehydrogenase [ubiquinone] 1 alpha subcomplex subunit 11	NDUAB (NDUFA11)
	OXPHOS	Q9UDW1	Cytochrome b-c1 complex subunit 9	UCR9 (UQCR10 UCRC, HSPC119)
	OXPHOS	P10606	Cytochrome c oxidase subunit 5B, mitochondrial	COX5B (COX5B)
	OXPHOS	P14927	Cytochrome b-c1 complex subunit 7	QCR7 (UQCRB UQBP)
	OXPHOS	P00403	Cytochrome c oxidase subunit 2	COX2 (MT-CO2 COII, COXII, MTCO2)
	OXPHOS	P24539	ATP synthase F(0) complex subunit B1, mitochondrial	ATP5F1 (ATP5F1)
	Muscle	O14958	Calsequestrin-2	CASQ2 (CASQ2)
	Muscle	Q6ZMU5	Tripartite motif-containing protein 72	TRI72 (TRIM72 MG53)
	Muscle	P10916	Myosin regulatory light chain 2, ventricular/cardiac muscle isoform	MLRV
	Muscle	P35609	Alpha-actinin-2	ACTN2 (ACTN2)
	Muscle	P08590	Myosin light chain 3	MYL3 (MYL3)
	Muscle	P13805	Troponin T, slow skeletal muscle	TNNT1 (TNNT1 TNT)
	Muscle	P13797	Plastin-3	PLST (PLS3)
	Muscle	P62736	Actin, aortic smooth muscle	ACTA (ACTA2 ACTSA, ACTVS, GIG46)
	Motor protein (binding ATP,NT)	P33176	Kinesin-1 heavy chain	KINH (KIF5B KNS, KNS1)
	Lipid metabolism	Q92523	Carnitine O-palmitoyltransferase 1, muscle isoform	CPT1B
	Lipid metabolism	P30084	Enoyl-CoA hydratase, mitochondrial	ECHM (ECHS1)
	Immune response	P40121	Macrophage-capping protein	CAPG (CAPG AFCP, MCP)
	Immune response	P62937	Peptidyl-prolyl cis-trans isomerase A	PPIA (PPIA CYPA)
	Glucose metabolism	Q7Z4W1	L-xylulose reductase	DCXR (DCXR SDR20C1)
	Glucose metabolism	P06733	Alpha-enolase	ENOA (ENO1 ENO1L1, MBPB1, MPB1)
	ETOH	P00325	Alcohol dehydrogenase 1B	ADHB1 (ADH1B ADH2)
	ETOH	P05091	Aldehyde dehydrogenase, mitochondrial	ALDH2 (ALDH2 ALDM)
	ECM	P00813	Adenosine deaminase	ADA (ADA ADA1)
	Developmental protein	Q16555	Dihydropyrimidinase-related protein 2	DPYL2 (DPYSL2 CRMP2, ULIP2)
	Binding protein (Ca2+)	P82279	Protein crumbs homolog 1	CRUM1 (CRB1)
	Binding protein (ATP,NT)	P05166	Propionyl-CoA carboxylase beta chain, mitochondrial	PCCB (PCCB)
	Amino acid biosynthesis	Q02252	Methylmalonate-semialdehyde dehydrogenase [acylating], mitochondrial	MMSA (ALDH6A1 MMSDH)
	Amino acid biosynthesis	Q9Y617	Phosphoserine aminotransferase	SERC (PSAT1 PSA)
	Amino acid biosynthesis	P17174	Aspartate aminotransferase, cytoplasmic	AATC (GOT1)

+ in Trained	Function	Uniprot	Description	
	Amino acid biosynthesis	Q53EW7	Branched-chain-amino-acid aminotransferase	Q53EW7 (BCAT2)
	Binding protein (Ca2+)	O75746	Calcium-binding mitochondrial carrier protein Aralar1	CMC1 (SLC25A12 ARALAR1)
	ECM	P17540	Creatine kinase S-type, mitochondrial	KCRS (CKMT2)
	ECM	P17540	Creatine kinase S-type, mitochondrial	KCRS (CKMT2)
	Glucose metabolism	P60174	Triosephosphate isomerase	TPIS (TPI1, TPI)
	Glucose metabolism	P04075	Fructose-bisphosphate aldolase A	ALDOA (ALDOA ALDA)
	Glucose metabolism	P04075	Fructose-bisphosphate aldolase A	ALDOA (ALDOA ALDA)
	Immune response	P12235	ADP/ATP translocase 1	ADT1 (SLC25A4 ANT1)
	Lipid metabolism	P09622	Dihydrolipoyl dehydrogenase, mitochondrial	DLDH (DLD GCSL, LAD, PHE3)
	Lipid metabolism	P40939	Trifunctional enzyme subunit alpha, mitochondrial	ECHA (HADHA HADH)
	Lipid metabolism	P42126	Enoyl-CoA delta isomerase 1, mitochondrial	ECI1 (ECI1 DCI)
	Lipid metabolism	P49753	Acyl-coenzyme A thioesterase 2, mitochondrial	ACOT2
	Muscle	P02144	Myoglobin	MYG (MB)
	NT homeostasis	P54819	Adenylate kinase 2, mitochondrial	KAD2 (AK2)
	OXPHOS	O43181	NADH dehydrogenase [ubiquinone] iron-sulfur protein 4, mitochondrial	NDUS4 (NDUFS4)
	OXPHOS	O75208	Ubiquinone biosynthesis monooxygenase COQ9, mitochondrial	COQ9 (COQ9)
	OXPHOS	O75489	NADH dehydrogenase [ubiquinone] iron-sulfur protein 3, mitochondrial	NDUS3 (NDUFS3)
	OXPHOS	O75947	ATP synthase subunit d, mitochondrial	ATP5H (ATP5H My032)
	OXPHOS	O75964	ATP synthase subunit g, mitochondrial	ATP5L (ATP5L)
	OXPHOS	P03915	NADH-ubiquinone oxidoreductase chain 5	NU5M (MT-ND5 MTND5, NADH5, ND5)
	OXPHOS	P31930	Cytochrome b-c1 complex subunit 1, mitochondrial	QCR1 (UQCRC1)
	OXPHOS	Q9P0J0	NADH dehydrogenase [ubiquinone] 1 alpha subcomplex subunit 13	NDUFA13 (NDUFA13 GRIM19, CDA016, CGI-39)
	OXPHOS	Q9Y2Z9	Ubiquinone biosynthesis monooxygenase COQ6, mitochondrial	COQ6 (COQ6 CGI-10)
	OXPHOS	Q9Y6M9	NADH dehydrogenase [ubiquinone] 1 beta subcomplex subunit 9	NDUB9 (NDUFB9 LYRM3, UQOR22)
	OXPHOS	Q16795	NADH dehydrogenase [ubiquinone] 1 alpha subcomplex subunit 9, mitochondrial	NDUA9 (NDUFA9 NDUFS2L)
	OXPHOS	P36542	ATP synthase subunit gamma, mitochondrial (F-ATPase gamma subunit)	ATPG (ATP5C1 ATP5C, ATP5CL1)
	OXPHOS	P28331	NADH-ubiquinone oxidoreductase 75 kDa subunit, mitochondrial	NDUS1 (NDUFS1)
	OXPHOS	P24539	ATP synthase F(0) complex subunit B1, mitochondrial	ATP5F1 (ATP5F1)
	OXPHOS	P24310	Cytochrome c oxidase subunit 7A1, mitochondrial	COX7A1 (COX7A1 COX7AH)
	OXPHOS	P14854	Cytochrome c oxidase subunit 6B1	COX6B1 (COX6B1 COX6B)
	OXPHOS	P00403	Cytochrome c oxidase subunit 2	COX2 (MT-CO2 COII, COXII, MTCO2)
	OXPHOS	P06576	ATP synthase subunit beta, mitochondrial	ATPB (ATP5B)
	OXPHOS	P03915	NADH-ubiquinone oxidoreductase chain 5	NU5M (MT-ND5 MTND5, NADH5, ND5)
	Porin	Q9Y2R7	Voltage-dependent anion-selective channel protein 3	VDAC3 (VDAC3)
	Skeletal muscle specific protein	Q96IX5	Up-regulated during skeletal muscle growth protein 5	USMG5 (USMG5 DAPIT, HCVFTP2, PD04912)
	Stress reponse	P04179	Superoxide dismutase [Mn], mitochondrial	SODM (SOD2)
	Stress reponse	Q9Y6C9	Mitochondrial carrier homolog 2	MTCH2 (MTCH2 MIMP, HSPC032)
	Stress reponse	P61604	10 kDa heat shock protein, mitochondrial	CH10 (HSPE1)
	Structure	Q00325	Phosphate carrier protein, mitochondrial	MPCP (SLC25A3 PHC, OK/SW-cl.48)
	Structure	Q02978	Mitochondrial 2-oxoglutarate/malate carrier protein	M2OM (SLC25A11 SLC20A4)
	TCA	O00330	Pyruvate dehydrogenase protein X component, mitochondrial	ODPX (PDHX PDX1)
	TCA	P07954	Fumarate hydratase, mitochondrial	FUMH (FH)
	TCA	P30086	Phosphatidylethanolamine-binding protein 1	PEBP1 (PEBP1 PBP, PEBP)
	TCA	P36957	Dihydrolipoyllysine-residue succinyltransferase component of 2-oxoglutarate dehydrogenase complex, mitochondrial	ODO2 (DLST)
	TCA	P48735	Isocitrate dehydrogenase [NADP], mitochondrial	IDHP (IDH2)
	TCA	Q02218	2-oxoglutarate dehydrogenase, mitochondrial	ODO1 (OGDH)
	TCA	Q99643	Succinate dehydrogenase cytochrome b560 subunit, mitochondrial	C560 (SDHC CYB560, SDH3)
	TCA	P31040	Succinate dehydrogenase [ubiquinone] flavoprotein subunit, mitochondrial	SDHA
	TCA	P30038	Delta-1-pyrroline-5-carboxylate dehydrogenase, mitochondrial	AL4A1 (ALDH4A1 ALDH4, P5CDH)
	TCA	P11177	Pyruvate dehydrogenase E1 component subunit beta, mitochondrial	ODPB (PDHB PHE1B)
	TCA	P21912	Succinate dehydrogenase [ubiquinone] iron-sulfur subunit, mitochondrial	SDHB (SDHB SDH, SDH1)

- in Trained	Function	Uniprot	Description	
	Stress reponse	P02511	Alpha-crystallin B chain	CRYAB (CRYAB CRYA2, HSPB5)
	Stress reponse	P11762	Galectin-1	LEG1 (LGALS1)
	OXPHOS	P20674	Cytochrome c oxidase subunit 5A, mitochondrial	COX5A (COX5A)

+ in OB/T2DM, - in Trained	Function	Uniprot	Description	
	**Muscle**	**Q96A32**	**Myosin regulatory light chain 2, skeletal muscle isoform**	**MLRS (MYLPF)**
	**Muscle**	**O14983**	**Sarcoplasmic/endoplasmic reticulum calcium ATPase 1**	**AT2A1 (SERCA1)**

- in OB/T2DM, + in Trained	Function	Uniprot	Description	
	**Lipid metabolism**	**P00505**	**Aspartate aminotransferase, mitochondrial**	**AATM (GOT2)**
	**OXPHOS**	**O75306**	**NADH dehydrogenase [ubiquinone] iron-sulfur protein 2, mitochondrial**	**NDUS2 (NDUFS2)**
	**OXPHOS**	**O95169**	**NADH dehydrogenase [ubiquinone] 1 beta subcomplex subunit 8, mitochondrial**	**NDUB8 (NDUFB8)**
	**OXPHOS**	**P06576**	**ATP synthase subunit beta, mitochondrial**	**ATPB (ATP5B)**
	**OXPHOS**	**P51970**	**NADH dehydrogenase [ubiquinone] 1 alpha subcomplex subunit 8**	**NDUA8 (NDUFA8)**

± in OB/T2DM, + in Trained	Function	Uniprot	Description	
	Glucose metabolism	P15259	Phosphoglycerate mutase 2	PGAM2 (PGAM2 PGAMM)
	Lipid metabolism	P00505	Aspartate aminotransferase, mitochondrial	AATM (GOT2)
	Muscle	P12882	Myosin-1	MYH1 (MYH1)
	OXPHOS	P25705	ATP synthase subunit alpha, mitochondrial	ATPA (ATP5A1 ATP5A, ATP5AL2, ATPM)

+ in OB/T2DM, + in Trained	Function	Uniprot	Description	
	TCA	O75390	Citrate synthase, mitochondrial	CISY (CS)
	TCA	P31040	Succinate dehydrogenase [ubiquinone] flavoprotein subunit, mitochondrial	SDHA
	TCA	Q99798	Aconitate hydratase, mitochondrial	ACON (ACO2)
	Stress reponse	P49411	Elongation factor Tu, mitochondria	EFTU (TUFM)
	Lipid metabolism	P49748	Very long-chain specific acyl-CoA dehydrogenase, mitochondrial	ACADV (ACADVL)
	Glucose metabolism	P15259	Phosphoglycerate mutase 2	PGAM2 (PGAM2 PGAMM)
	ECM	P02765	Alpha-2-HS-glycoprotein	FETUA (AHSG FETUA, PRO2743)
	Binding protein (ATP, NT)	Q9HCC0	Methylcrotonoyl-CoA carboxylase beta chain, mitochondrial	MCCB (MCCC2)

- in OB/T2DM, - in Trained	Function	Uniprot	Description	
	**Glucose metabolism**	**P15121**	**Aldose reductase**	**ALDR (AKR1B1 ALDR1)**
	**Lipid metabolism**	**Q8N335**	**Glycerol-3-phosphate dehydrogenase 1-like protein**	**GPD1L (GPD1L KIAA0089)**

± in OB/T2DM only	Function	Uniprot	Description	
	Binding protein (FE2+,Fe3+, O2, heme)	P69905	Hemoglobin subunit alpha	HBA (HBA1 HBA2)
	Lipid metabolism	P02647	Apolipoprotein A-I	APOA1 (APOA1)
